# Deletion of the major *Escherichia coli* multidrug transporter *AcrB* reveals transporter plasticity and redundancy in bacterial cells

**DOI:** 10.1371/journal.pone.0218828

**Published:** 2019-06-28

**Authors:** Noémie Alon Cudkowicz, Shimon Schuldiner

**Affiliations:** Department of Biological Chemistry, Institute of Life Sciences, Hebrew University of Jerusalem, Givat Ram, Jerusalem, Israel; Institut National de la Recherche Agronomique, FRANCE

## Abstract

Multidrug Transporters (MDTs) are major contributors to the acquisition and maintenance of Antimicrobial Resistance (AMR), a growing public health threat of broad concern. Despite the large number of MDTs, the overwhelming majority of the studies performed thus far in Gram-negative bacteria emphasize the supremacy of the AcrAB-TolC complex. To unveil the potential role of other MDTs we studied the behavior of a null AcrB *Escherichia coli* strain when challenged with chloramphenicol, a bacteriostatic antibiotic. We found that such a strain developed an extremely high-level of resistance to chloramphenicol, cross resistance to quinolones and erythromycin and displayed high levels of expression of the single component MFS transporter MdfA and multiple TolC-dependent transporters. The results suggest that the high versatility of the whole ensemble of transporters, the bacterial Effluxome, is an essential part of a strategy of survival in everchanging, at times noxious, environments. The concept of a functional Effluxome presents an alternative to the existing paradigms in the field and provides novel targets for the search for inhibitors of transporters as adjuvants of existing antibiotics.

## Introduction

Multidrug Transporters (MDTs) are major contributors to the acquisition and maintenance of Antimicrobial Resistance (AMR). MDTs comprise a large group of ubiquitous polyspecific transporters that recognize a wide range of dissimilar substrates that may differ in structure, size or electrical charge, and actively remove them from the cell [1–4]. In a model organism such as *Escherichia coli* more than 20 different MDTs have been shown to confer resistance to one or more drugs when overexpressed [5]. Nevertheless, the overwhelming majority of the studies performed thus far support the central role of AcrAB-TolC complex (see for example [4, 6–9]. A systematic assessment of the role of specific genes in resistance to antibiotics and in terms of transporter genes only identified the effect of the AcrAB-TolC complex and nothing else [10]. It seems that the contribution of other transporters escaped detection because of backup compensation in which transporters with overlapping functionality cover for the loss of the other ones [11–14]. In Gram Negative bacteria, AcrAB-TolC is responsible for removal out of the cell of solutes such as drugs before entering the cell or after they were actively transported from the cytoplasm away from their targets by single component transporters in the plasma membrane [4, 11–15].

We previously found that, in the process of acquisition of resistance to norfloxacin, a widely used bactericidal antibiotic, a knockout of AcrB surprisingly developed a resistance almost as high as the one reached by wild type cells [11]. Expression levels of AcrEF-TolC, a very close homologue of AcrAB-TolC, increased significantly and provided an excellent backup to the null mutant strain [11]. To further explore this versatility of the transporters ensemble and to test the generality of the phenomenon we analyze here the behavior of a null AcrB strain when challenged with chloramphenicol, a bacteriostatic antibiotic with a very different mode of action. As previously reported, such a strain, devoid of AcrB, developed an extremely high-level of resistance to chloramphenicol [11]. Here we study this strain in detail and show that it displays high levels of expression of the single component MFS transporter MdfA and multiple TolC-dependent transporters. The results support the notion that in any living cell dozens of transporters labor together to maintain a very accurate homeostatic environment, their interaction and crosstalk dictated by thermodynamics and regulated when needs arise. Exposure to a stressor such as an antibiotic brings about global and major changes in metabolism and to a new equilibrium in the cellular composition that necessitates changes in the activity of specific transporters. Efforts to develop specific inhibitors of transporters as adjuvants to existing antibiotics must also take into consideration the high versatility of the whole ensemble of transporters, the bacterial Effluxome.

## Materials and methods

### Strains and plasmids

*E*. *coli* BW25113 strain was used throughout this work. BW25113 Δ*emrE*, Δ*mdfA*, Δ*mdtM* and Δ*acrB*, individual knockouts and combinations were described by Tal and Schuldiner [12] and prepared essentially according to Datsenko and Wanner [16, 17]. After generation of the knockouts, the kanamycin resistance gene was excised as described [17]. The plasmids used for gene expression are pT7-7, pT7-5 [18] and pKK223-3 (PL-Pharmacia).

### Evolution process

The *in-vitro* evolution process was carried out as previously described by Shuster and collaborators [11]. In brief, the *E*. *coli* cells were grown overnight in 20 mL LB-KP_i_ medium (LB medium containing 70 mM potassium phosphate, pH 7.4) containing 0.1μM chloramphenicol, a sub-lethal concentration. Cultures that reached at least A_600_ = 1 were further diluted 1:100 to LB-KP_i_ containing twice the chloramphenicol concentration, grown overnight yet again and examined for growth. The process was continued with a daily two-fold increase in the chloramphenicol concentration. In case the culture did not reach A_600_ = 1 after overnight growth, the cells were allowed to grow one more night. If no growth was recorded, the cells were moved to a previous, lower concentration of chloramphenicol and allowed to grow again. The sequential increase was stopped if the cells reached a concentration in which no growth was recorded after 48 hours.

Throughout the evolution process, the cultures were tested daily using PCR to verify the stability of the acrB and the triple knockouts. Primers complementary to chromosomal sequences flanking the knockout regions were used and the size of the product was compared to that of the wild type strain and the calculated value for each given knockout (S3 Table). At the end of the experiment individual colonies were isolated on LB-CAM (200μM) and tested again for resistance. Finally, one clone was selected and chromosomal DNA was prepared using the NucleoSpin® Tissue kit (Macherey&Nagel, Düren, Germany). The libraries were prepared with Nextera XT DNA Library Prep kit (Illumina) using the extracted genomic DNA, and sequenced using 150-bp paired-end reads on the Illumina NextSeq500 platform. The results were analyzed using the Geneious 11.0.5 software. The assembly and mapping was performed against *Escherichia coli* BW25113 complete genome, accession CP009273.1 from the NCBI website. The genome was then aligned to the same reference genome using the Mauve plugin [19] to identify the potential mutations. To validate each one of them, DNA segments of about 1kb around the mutation in question were amplified by PCR and sequenced using one of the primers that had served for the PCR amplification (S4 Table). Once the mutation was confirmed for the end-point evolved strain, we used the same process and checked colonies isolated from the days along the process, to pinpoint the exact day on which the mutation had been fixed.

### Determination of resistance levels in liquid medium

For resistance in liquid medium, overnight cultures were diluted into LB medium, grown to early logarithmic phase, and diluted to A_600_ = 0.01 in LB-KP_i_ medium at the indicated concentrations of toxic compounds in a 96-well plate (Nunc) at 37°C with constant shaking. A_600_ readings were taken every hour for 12 h using a Synergy 2 Microplate Reader (BioTek). Plots of bacterial growth vs. drug concentrations were then fitted using OriginLab’s Origin Pro 9.1 software. For each time point the IC_50_ value was determined at least three times.

### Determination of resistance levels on solid medium

For testing resistance on solid medium, cells were grown overnight at 37°C in LB medium. The cultures were diluted to A_600_ = 0.2, and 5 μL of serial dilutions of the culture were spotted onto LB plates containing 70 mM KP_i_, pH 7.4, with or without the addition of the indicated concentrations of antibiotic. Growth was visualized after overnight incubation at 37°C. In all cases growth of all the strains in the absence of antibiotics was similar. All experiments were repeated at least twice.

The disc assay method gives us yet another way to visualize bacterial resistance to different types of antibiotics. Overnight cultures of bacteria are mixed with soft agar medium (0.6% agar) and spread on top of regular LB plates. The bacteria are then allowed to grow for 2 hours while antibiotic discs are prepared: a small volume (between 20 and 30 μl) of a set concentration of antibiotic is soaked into sterile paper disc and allowed to dry. The discs are then placed on the surface of the plates containing the bacteria, and these are incubated overnight.

### Determination of transcript levels

The bacterial total RNA was extracted using PureLink® RNA Mini Kit (Ambion) and cDNA was synthesized from 1.0 μg of RNA using the High Capacity RNA-to-cDNA™ kit (Applied Biosystems™) and stored at -80°C.

The cDNA levels of target genes were determined by relative quantitative real time PCR using the StepOne Plus Real Time PCR system (Applied Biosystems) in a 20 μl reaction for 40 cycles. Reactions were prepared using the Fast SYBR® Green Master Mix the primers shown in S5 Table. Analysis of gene expression was performed using the comparative C_T_ method [20], normalized to the control *gapDH* gene (AKA *gapA*) coding for the glyceraldehyde 3-phosphate dehydrogenase.

## Results

### *Escherichia coli* acquires high-level resistance to chloramphenicol

We previously reported the generation in the lab of *E*. *coli* strains highly resistant to chloramphenicol through multistep evolution by exposing the cells to sublethal concentration of the drug, increasing it daily two-fold until the bacteria were no longer able to adapt to the amount of chloramphenicol [11]. In this study we wanted to evaluate the role of MDTs in the process of adaptation. We used two types of mutants: a triple single-component (EmrE, MdfA and MdtM) knock out strain, and a single tripartite transporter (AcrB) knock out strain. These strains provide an excellent experimental paradigm to analyze the potential role of transporters other than the AcrAB-TolC complex and to try to pinpoint regulatory mechanisms governing the interaction of MDTs in a Gram-Negative organism such as *E*. *coli*. We here offer a detailed evaluation of the phenotype of above strains, identify chromosomal mutations and evaluate expression levels of various transporters by quantitation of transcript levels.

**Fig 1:**
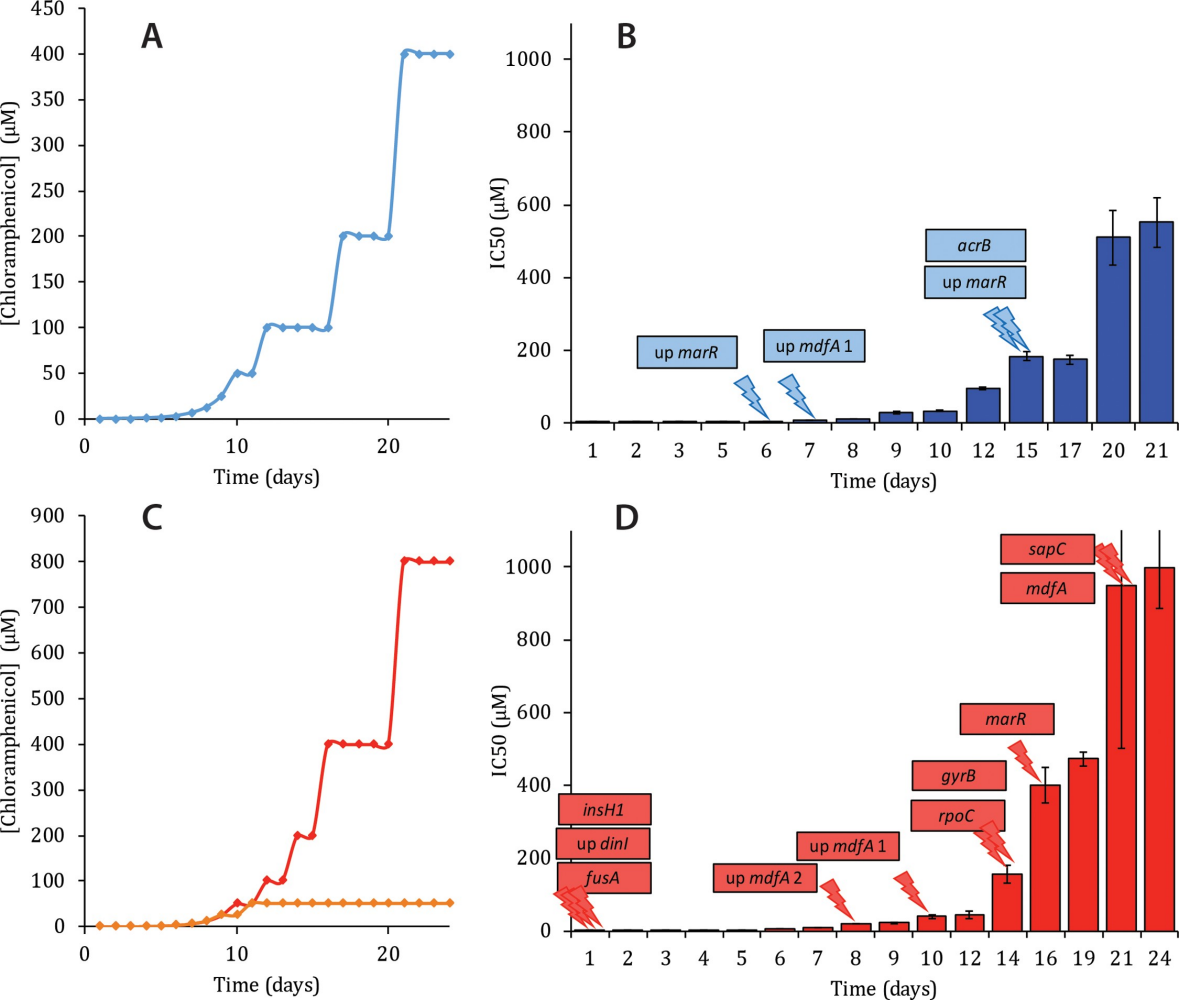
Time course for the evolution of the high-level resistance to chloramphenicol. **A**. and **C**. Cells were grown overnight in the presence of 0.1 μM chloramphenicol and cultures that reached at least A_600_ = 1 were further diluted to LB-KPi containing twice the chloramphenicol concentration. A. BW25113; C. BW25113 Δ*emrE*, Δ*mdfA*, Δ*mdtM* (orange) and Δ*acrB* (red) **B**. and **D**. For each day of the process, the IC_50_ value was determined using a liquid phenotype assay as described under Materials and Methods. In **1B** the IC_50_ value for wild type BW25113 increases gradually with time, with a sharp increase between days 17 and 20. The IC_50_ eventually reaches a value of 550 μM chloramphenicol (+/- 68 μM), having started at a resistance value of 3.9 μM (+/- 0.3 μM), an increase of about 140-fold in resistance. In **1D** the increase in IC_50_ values for the Δ*acrB* strain is dramatic from 0.5 μM to 1 mM, an increase of 2000 fold (for individual values see also S1 Table). The appearance of the mutations is indicated for each strain in 1B and 1D correspondingly.

Cells originated from *E*. *coli* BW25113 cells (naïve wild type) subjected to the in-lab evolution protocol above described are able to grow at concentrations as high as 400 μM (Fig 1A), 4000 times higher than the concentration at the onset of the experiment. A clone isolated at the end of the experiment is named EVC (**EV**olved in **C**hloramphenicol) and displays an IC_50_ of approximately 500 μM (see S1 Table for specific values of IC_50_). The strain with a triple null mutation of *emrE*, *mdfA* and *mdtM* was only able to achieve a medium-level resistance to chloramphenicol and could not grow at concentrations higher than 50 μM (Fig 1C). On the other hand, cells carrying a null mutation of *acrB* were able to grow on the extremely high concentration of 800 μM of chloramphenicol, even higher than the EVC strain (Fig 1C). The resistant *ΔacrB* strain, *ΔacrB* EVC, displays an IC_50_ of around 1 mM, a remarkable 2000-fold increase compared to the naïve *ΔacrB* strain (S1 Table).

### Identification of mutations in the resistant strains

The stress induced by exposure of cells to noxious agents such as antibiotics has a dual effect. In parallel to a transcriptional activation of expression of MDTs, stress induced mutagenesis increases the mutability in the population. The likelihood of mutations that confer advantage to be fixed is amplified when the MDTs actively remove some of the offending compound and thereby allow the cells bearing that mutation to rapidly multiply. Such a sub-population of bacteria may then accumulate additional mutations with prolonged exposure to a constant or increasing concentration of antibiotic [9, 11, 21–25].

To identify such mutations we performed whole genome sequencing on the resistant strains isolated in the lab.

In the EVC strain, we observed a rather small number of mutations: one single nucleotide mutation in the gene *acrB*, coding for the membrane subunit of the AcrAB-TolC multidrug efflux transporter (V139F), a distal binding pocket mutation, probably altering the binding of specific compounds [26]; a single nucleotide mutation (C to A) located 12 nucleotides upstream of *mdfA*; and two mutations upstream of the gene encoding for the DNA-binding transcriptional repressor MarR–one single nucleotide deletion 65 nucleotides upstream of its start codon, and a deletion of 20 nucleotides, ending 7 bases upstream of the start codon (S2 Table). As seen in Fig 1B, the first mutation to appear upon exposure to chloramphenicol is the single nucleotide insertion upstream of *marR* on day 7 of the evolution process, then on day 8 the mutation upstream of *mdfA* is detected, and lastly on day 15 appear both the mutations in *acrB* and the deletion upstream of *marR*.

The *ΔacrB* EVC strain–which acquires a strikingly high level of resistance to chloramphenicol–presented a larger number of mutations after the evolution process (Fig 1D). Three mutations appear already on day 1 of the process: the insertion of the *insH1* gene (the transposase for the insertion sequence element *IS5*), upstream of the gene *uspC*, coding for universal stress protein C; a single-nucleotide mutation in *fusA*, coding for elongation factor G which facilitates the translocation of the ribosome along the mRNA molecule (G516S); and a single-nucleotide mutation upstream of *dinI* (T to C, 27 bp upstream of the gene), coding for DNA-damage inducible protein I. On day 8, appears a first mutation upstream of *mdfA*, a single-nucleotide change from T to G, 57 nucleotides upstream of the gene. On day 10, another single nucleotide mutation can be identified upstream of *mdfA*, interestingly the same mutation that was conserved by the EVC as well, C to A, 12 nucleotides upstream of the gene. On day 14, as the IC_50_ of the strain is increased 3-fold, from about 42 μM to 155 μM, two new mutations are detected: one in the gene *gyrB* coding for DNA gyrase subunit B (H237R), and another in the gene *rpoC*, coding for RNA polymerase subunit β’ (F338C). The mutation in *marR* appears on day 16: starting at nucleotide 311 of the gene, 12 nucleotides are deleted. Lastly, on day 21 appear two mutations in genes coding for transporter proteins: one in *mdfA*, L156V, and one in *sapC*, coding for a transmembrane protein part of the ATP Binding Cassette superfamily, in which a stop codon is formed 24 nucleotides after the beginning of the gene (S2 Table). SapC is one of two integral membrane subunits of a predicted transporter complex, SapBCDF, which has been implicated in putrescine efflux [27].

Remarkably, two mutations detected here were previously reported in Norfloxacin resistant strains [11]. The *fusA* mutation identified here in *ΔacrB EVC* (G516S) at the very onset of the in-lab evolution protocol is identical to the one previously reported when *ΔacrB* cells were exposed to Norfloxacin [11]. Moreover, the mutation in *rpoC* reported here (F338C) is very close to that reported for the Norfloxacin exposed cells (D348A). Interestingly, *gyrB*, a target of fluoroquinolones, is also mutated upon exposure to Chloramphenicol. Despite the difference in the mechanism of action of two antibiotics, the bactericidal Norfloxacin and the bacteriostatic Chloramphenicol, this overlap suggests common pathways of response to stress.

The role of these and the other mutations detected in the high-level resistance strain needs to be established and further analysis should enlighten the multiple survival strategies that allow bacteria to adapt to challenging environments. Besides the *mdfA* and *marR* associated mutations, none of the others above described have, to our knowledge, been previously associated with chloramphenicol resistance and their possible role deserves further investigation.

### The resistant strains undergo important transcriptional changes in the transporter ensemble

In addition to the identification of mutations in resistant strains, we evaluated in EVC and Δ*acrB* EVC transcript levels of genes whose expression we presumed to be involved in the acquisition and maintenance of resistance to chloramphenicol.

**Fig 2:**
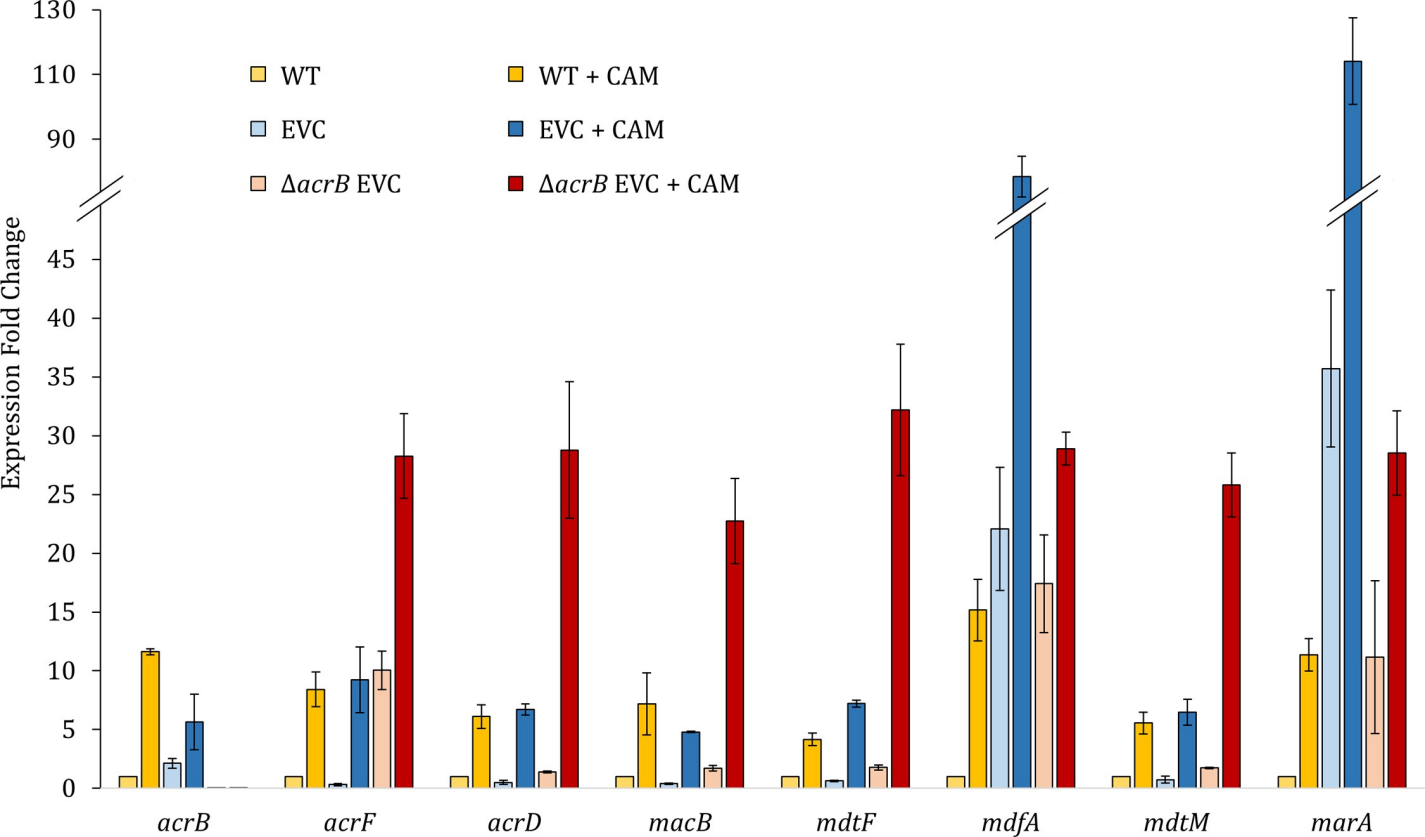
Expression of TolC dependent transporters increases during evolution in the Δ*acrB* strain. The levels of RNA transcripts of the genes coding for the TolC dependent transporters AcrB, AcrD AcrF, MdtF and MacB, the MFS transporters MdfA and Mdtm and the regulator MarA were determined in EVC and Δ*acrB* EVC. The increase in transcripts of *mdfA* and *marA* are out of scale and present a fold change of 78-fold and 114-fold in Δ*acrB* EVC + CAM (CAM: chloramphenicol), respectively; the chart was adapted for clarity. Error bars represent the standard deviation. WT + CAM strain was grown with 5 μM chloramphenicol, EVC + CAM and ΔacrB EVC + CAM with 400 μM.

We detected a dramatic increase in the transcript levels of *mdfA* (Fig 2), most likely, the result of the mutation upstream of *mdfA*. The mutations in *marR* resulted in inactivation of this repressor that resulted in a ~100 fold increase in the transcript levels of *marA* (Fig 2), a well-known transcriptional regulator of a large number of genes involved in resistance to antibiotics, oxidative stress, organic solvents, and heavy metals [28, 29]. Similar to the EVC strain, the *mdfA* and *marR* associated mutations in the *ΔacrB* EVC strain led to dramatic increases in the transcript levels of *mdfA* and *marA* (Fig 2).

Transcript levels of the TolC transporters, AcrE, AcrD, MacB, MdtF correlate with the expression and/or activity of *acrB*. In the EVC strain, where the AcrB transporter is functional, expression of *acrF*, *acrD*, *macB* and *mdtF* is repressed relative to the naïve strain when grown without chloramphenicol, (S1 Fig) and is only partially relieved when cells are grown on chloramphenicol (Fig 2). On the other hand, in the null *acrB* strain, the basal levels are higher than in the naïve strain (S1 Fig), and increase dramatically when cells are grown in the presence of chloramphenicol (Fig 2). The increase in expression ranged from 4 (*mdtF* in the naive WT strain) to about 80-fold (*mdfA* in the EVC strain).

### Cross-resistance to other antibiotics

The increase in expression of the TolC-dependent transporters AcrB, AcrE, AcrD, MacB, MdtF and the activation of the Mar regulon brought about by the higher level of the transcriptional activator MarA, could lead to resistance to other antibiotics as well. Cross-resistance to an antibiotic following exposure and acquisition of resistance to a different drug has been reported in the past [6, 30]. We therefore set out to check if the chloramphenicol resistant strains isolated here had also become resistant to drugs from other families. For this purpose, we performed disc assays. Four strains were used for these experiments: naïve WT, naïve Δ*acrB*, EVC and Δ*acrB* EVC and the assay was performed on plates containing chloramphenicol at concentrations appropriate for the specific strain (1 μM for the naïve strains, 100 μM for the resistant).

**Fig 3:**
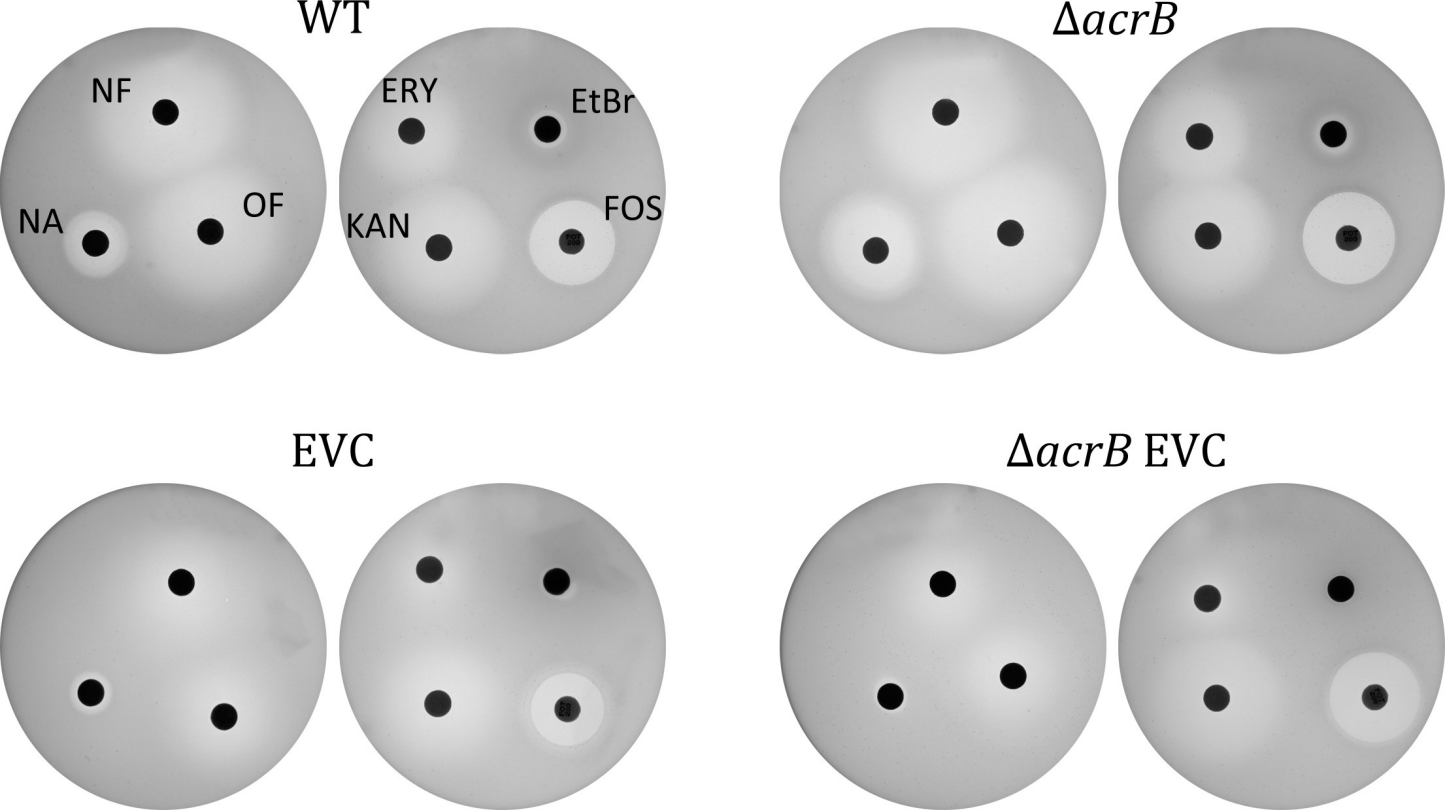
Strains selected for resistance to chloramphenicol display cross-resistance to other antibiotics. The disc assay was used to test resistance to NF, norfloxacin; OF, ofloxacin; NA, nalidixic acid; ERY, erythromycin; EtBr, ethidium bromide; KAN, kanamycin and FOS, fosfomycin. For sake of clarity, the order is indicated only in one of the strains.

As seen in Fig 3, both EVC strains, and particularly the Δ*acrB* EVC, display an increased resistance to the quinolone antibiotics nalidixic acid, ofloxacin and norfloxacin. Moreover, we can see that the resistance to ethidium bromide and erythromycin is increased as well in the resistant strains. There are only small effects on the sensitivity to kanamycin and fosfomycin.

Thus, as previously reported for norfloxacin and other antibiotics [6, 11, 30], strains selected for resistance on one antibiotic, in our case chloramphenicol, display resistance to multiple ones. The cross resistance can be, at least, partially explained by the increased expression of MDTs with different but overlapping specificities.

### Maintenance of high-level resistance to chloramphenicol is dependent on MDTs

**Fig 4:**
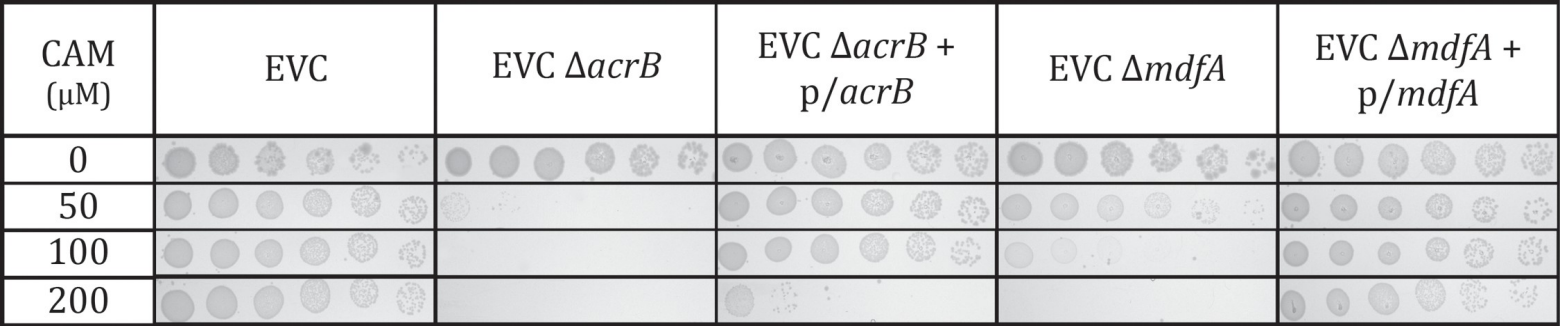
The role of the transporters in maintenance of high-level resistance. Serial dilutions of cells were spotted on plates supplemented with the indicated concentrations of chloramphenicol. Shown are the results for EVC and the corresponding null mutations of *acrB* and *mdfA* and their complementation. The control plasmid was pT7-7, *mdfA* was expressed from pT7-5 and *acrB* from pKK 223–3.

We showed above the role of MDTs in the process of acquisition of resistance. Next we ask whether they have also a role in maintenance of the resistance, once all the mutations were fixed. While bacteria are able to acquire high-level resistance to chloramphenicol even without the main RND-type multidrug efflux transporter AcrB, in a strain with a functional AcrB that has acquired the high-level resistance (EVC), knocking out *acrB* greatly diminishes its resistance to chloramphenicol (Fig 4). The higher susceptibility was indeed caused by the null mutation since introduction of an acrB-expressing plasmid restored the resistance (Fig 4).

As discussed above, knockout mutants of MDTs other than AcrB were usually indistinguishable from the wild type counterpart regarding sensitivity to antibiotics [10, 31]. Also in the case of the *mdfA* gene knocking it out by itself in *E*. *coli* W3110, a naïve wild type strain, did not affect much the capability of the bacteria to resist to chloramphenicol and other antibiotics [10, 31]; a decreased resistance was observed only when a knockout was generated in a mutant with an impaired outer membrane [32]. Interestingly, however, here we show that after the bacteria have become resistant, deleting *mdfA* causes a notable increase in the susceptibility (Fig 4). As shown for the *acrB* null mutation, the higher susceptibility was indeed caused by the null mutation since the resistance was restored when *mdfA* was reintroduced on a plasmid (Fig 4).

## Discussion

Multidrug efflux transporters (MDTs) are a ubiquitous class of integral membrane proteins of vast clinical interest because of their strong association with human disease and pharmacology [2, 6, 33, 34]. The results described in this paper provide further support to their role in an organism such as *E*. *coli* and to the strong protective network that is achieved by a high versatility of expression and function. The adaptability of the network shown here underscores the role of individual transporters in response to stressors such as the bacteriostatic antibiotic chloramphenicol and, as previously shown, the bactericidal quinolones [11].

High-level resistance to chloramphenicol has usually been associated with the activity of the chloramphenicol acetyl transferase (CAT), encoded by the *cat* gene, that inactivates it by covalently linking one or two acetyl groups to the hydroxyl groups in the molecule [35]. Resistance as a consequence of target site mutation is rarely seen but lower membrane permeability and activity of efflux transporters have been also linked to resistance in multiple species [35].

To unveil the role of multidrug transporters other than AcrB in the process of acquisition and maintenance of high-level resistance, we studied the performance of a null *acrB* mutant. The response of this mutant provided a unique opportunity to highlight the activity of other TolC-dependent transporters: when the null *acrB* mutant is exposed to chloramphenicol, increase in transcription levels of AcrF, AcrD, MacB and MdtF, provide a strong backup. AcrF, AcrD and MdtF are part of the RND family of transporters, are highly homologous to the AcrAB multidrug efflux system and their substrate specificity shows considerable overlap [5]. Although *acrEF* is not expressed at significant levels in wild-type K-12 strains [36] overexpression of cloned *acrEF* in a strain which lacks AcrB confers increased resistance to a large number of antibiotics and toxicants [5]. Upregulation has been reported in null *acrB* mutants upon exposure to quinolones [11, 37] and also by integrational activation of the *acrEF* operon with insertional elements in *E*. *coli* [38, 39] and S. typhimurium [40]. Individual deletions of *mdtEF*, *acrEF*, *acrD*, *macB* in the K-12 strain W3110 did not affect susceptibility to a range of 35 compounds (dyes, detergents, antibiotics and others) [31].

MdfA and MdtM are well-characterized H^+^-drug antiporters from the MFS superfamily [41–43]. The results shown here support their well-documented role in resistance to chloramphenicol [5, 44]. The fact that the triple null mutant including *mdfA*, *mdtM* and *emrE* did not develop high-level resistance support their role in the acquisition process. Moreover, the increased transcript levels of the genes coding for both transporters and the susceptibility displayed by the EVC null *mdfA* support also its role in the maintenance of the high-level resistance.

Much is known about the regulation of expression of the AcrAB-TolC complex and the central role played by the global regulators MarA, SoxS and Rob [3, 29]. The mechanism that mediates the concerted increase in transcription of the various TolC transporters observed here in the absence of AcrB needs further elucidation. In *Salmonella enterica* a coordinated response of several transporters was detected by activation of *ramA* and *marA* [45]. In our case, an increase in *marA* expression alone does not provide a full explanation since the increase in *marA* transcripts was even more dramatic in the EVC strain than in the Δ*acrB* EVC. Despite this increase, transcript levels of *acrF*, *acrD*, *macB* and *mdtF* were lower than in the naïve strain. Several published reports suggest MarA independent regulation of expression of some but not all the above transporters. Thus, deletion of *hns* was previously shown to increase the resistance of a null *acrB* strain to antibiotics, dyes, antiseptics and detergents due to increased expression of the drug exporters AcrEF and MdtEF but not AcrD, MacB and others tested [46]. Also, the EvgA response regulator was shown to induce overexpression of MdtEF expression [47] and the PhoPQ two-component system was proposed as a regulator of expression of MacB [48].

Further studies are needed for detailed elucidation of the concerted regulation of all the tested TolC-dependent transporters, a regulation that provides a very robust backup response that may pose a serious obstacle for design of efflux pumps inhibitors (EPI) based antibiotic adjuvants [49–51].

We anticipate that the concept of an effluxome where each member participates in the removal of noxious chemicals from the cell should contribute to improve the present strategy searching for transport inhibitors as adjuvants of existing antibiotics and provide novel targets for this urgent undertaking. The process of identification of its members and the elucidation of the nature of the interactions will throw novel light on the roles of transporters in bacterial physiology and development of drug resistance.

## Supporting information

S1 Fig Expression of TolC dependent transporters increases during evolution in the Δ*acrB* strain. (PDF)

S1 Table IC_50_ values determined during the evolution process. (PDF)

S2 Table Summary of the mutations detected in the EVC and ΔacrB EVC strains. (PDF)

S3 Table Primers used for validation of knock-out stability. (PDF)

S4 Table Primers used for validation of mutations and determination of their appearance in the evolution process. (PDF)

S5 Table Primers used for the determination of transcript levels. (PDF)

## Abbreviations

MDTs: Multidrug Transporters
AMR: Antimicrobial Resistance
MFS: Major Facilitator Superfamily
CAM: Chloramphenicol
