# Supplementary material for: Deletion of the major Escherichia coli multidrug transporter AcrB reveals transporter plasticity and redundancy in bacterial cells
Source: PLoS One. 2019 Jun 28;14(6):e0218828. doi: 10.1371/journal.pone.0218828 (PMC6599122; doi:10.1371/journal.pone.0218828)
Supplement: S1 Fig — (PDF) [file pone.0218828.s001.pdf]

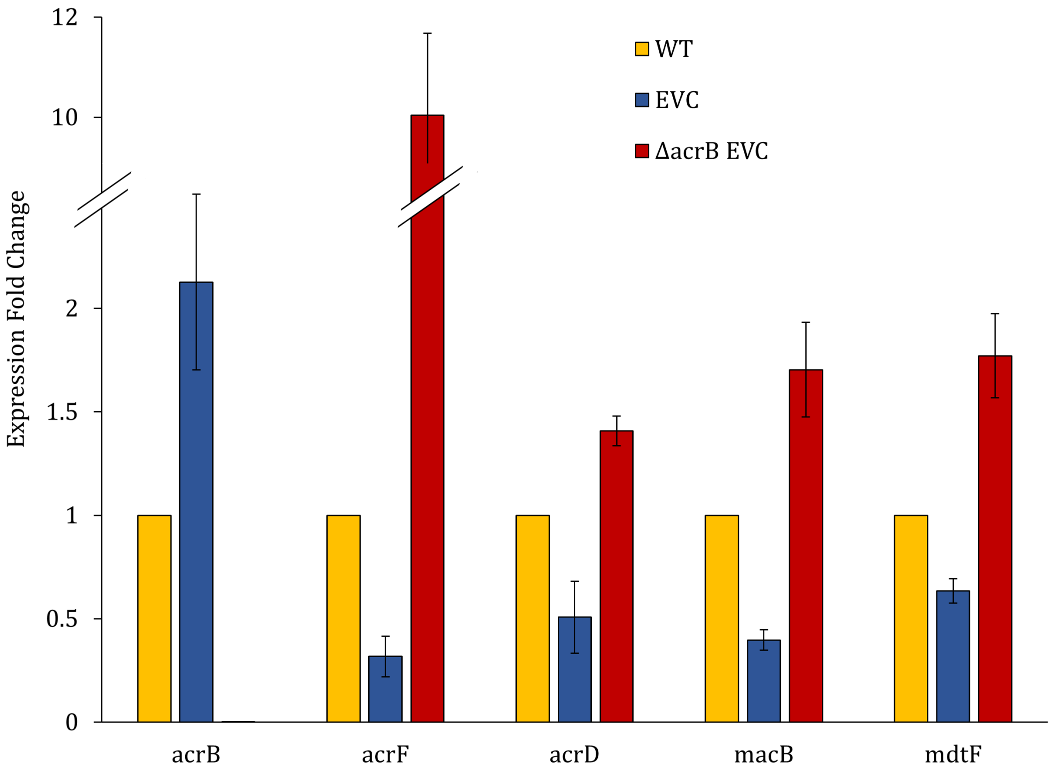

Figure S1: Expression of TolC dependent transporters increases during evolution in the Δ*acrB* strain. To emphasize the differences in the low range of transcript levels, only the results without Chloramphenicol from Figure 2 are shown and the scale is modified accordingly.
