# Supplementary material for: Deletion of the major Escherichia coli multidrug transporter AcrB reveals transporter plasticity and redundancy in bacterial cells
Source: PLoS One. 2019 Jun 28;14(6):e0218828. doi: 10.1371/journal.pone.0218828 (PMC6599122; doi:10.1371/journal.pone.0218828)
Supplement: S1 Table — (PDF) [file pone.0218828.s002.pdf]

Day	EVC	ΔacrB EVC
**1**	3.93 ± 0.27	0.52 ± 0.17
**2**	3.76 ± 0.14	0.42 ± 0.09
**3**	4.60 ± 0.58	0.46 ± 0.06
**4**	nd	0.37 ± 0.03
**5**	3.48 ± 0.17	2.89 ± 0.46
**6**	3.89 ± 0.16	6.79 ± 0.18
**7**	6.96 ± 0.22	7.76 ± 0.61
**8**	10.44 ± 0.41	19.12 ± 0.72
**9**	28.65 ± 2.94	21.16 ± 2.91
**10**	33.55 ± 0.75	39.06 ± 5.88
**12**	94.80 ± 4.60	42.68 ± 10.63
**14**	nd	155.63 ± 25.74
**15**	184.04 ± 12.42	nd
**16**	nd	400.54 ± 50.16
**17**	173.92 ± 11.64	nd
**19**	nd	472.23 ± 20.30
**20**	510.75 ± 75.25	nd
**21**	551.55 ± 68.79	947.55 ± 445.55
**24**	nd	998.26 ± 112.52

Table S1: IC_{50} values determined during the evolution process.

IC_{50} values, in µM, are the average of at least 3 replicas. Nd, not determined.
