# Supplementary material for: Deletion of the major Escherichia coli multidrug transporter AcrB reveals transporter plasticity and redundancy in bacterial cells
Source: PLoS One. 2019 Jun 28;14(6):e0218828. doi: 10.1371/journal.pone.0218828 (PMC6599122; doi:10.1371/journal.pone.0218828)
Supplement: S2 Table — (PDF) [file pone.0218828.s003.pdf]

	Gene	DNA change	Protein change
**EVC**	*acrB*	415G>T	Val139Phe
	upstream *mdfA1*	(-12)C>A	-
	upstream *marR*	(-27)_(-8)del	-
	upstream *marR*	(-65)delA	-
**Δ*acrB* EVC**	upstream *mdfA2*	(-57)T>G	-
	upstream *mdfA1*	(-12)C>A	-
	mdfA	466C>G	Leu156Val
	upstream *dinI*	(-27)T>C	-
	sapC	25G>T	Glu9Stop
	marR	311_322del	Gly104Stop
	upstream *uspC*	(-331)A>insertion of insH1	-
	fusA	1546G>A	Gly516Ser
	gyrB	710A>G	His237Arg
	rpoC	1013T>G	Phe338Cys

Table S2: Summary of the mutations detected in the EVC and ΔacrB EVC strains
