# Supplementary material for: Deletion of the major Escherichia coli multidrug transporter AcrB reveals transporter plasticity and redundancy in bacterial cells
Source: PLoS One. 2019 Jun 28;14(6):e0218828. doi: 10.1371/journal.pone.0218828 (PMC6599122; doi:10.1371/journal.pone.0218828)
Supplement: S3 Table — (PDF) [file pone.0218828.s004.pdf]

Primer	Sequence
acrB L1	5′ TGATCACCAGTGACGGCATT 3′
acrB L2	3′ GCGGGACAGGACTCACTTTT 5′
mdfA L1	5′ AGCCAATACAGGCAAGTCGT 3′
mdfA L2	3′ AATGCCTGATCGCACAAAGC 5′
emrE L1	5′ AAGGTCTGGCGAACGGTGTATT 3′
emrE L2	3′ GTGACACCTGCTAACGTATGCT 5′
mdtM L1	5′ TGTTCACAGGCAGTGTGGTT 3′
mdtM L2	3′ GCGTAACGACAAAGGTAGCA 5′

Table S3. Primers used for validation of knock-out stability
