# Supplementary material for: Deletion of the major Escherichia coli multidrug transporter AcrB reveals transporter plasticity and redundancy in bacterial cells
Source: PLoS One. 2019 Jun 28;14(6):e0218828. doi: 10.1371/journal.pone.0218828 (PMC6599122; doi:10.1371/journal.pone.0218828)
Supplement: S5 Table — (PDF) [file pone.0218828.s006.pdf]

Gene	Forward primer	Reverse primer
acrB	5′ GAAGAGCACGCACCACTACAC 3′	3′ GCAGACGCACGAACAGATAGG 5′
acrF	5′ GCGCGTGATGATGGAGGATA 3′	3′ AAATACCGCTGACAGCACCA 5′
acrD	5′ AGCGGTCTTGGTAGTTCTGC 3′	3′ CGCCAGCAACTGATTACGTG 5′
macB	5′ GGCTGGAAGACCGTACAGAG 3′	3′ GTTGGTTCATCGGCAAGAAT 5′
mdfA	5′ CCACGCGTATAGGCGAGAAA 3′	3′ CGGCAGACTAACGAATCCCA 5′
marRAB	5′ CATAGCATTTTGGACTGGAT 3′	3′ TACTTTCCTTCAGCTTTTGC 5′
mdtM	5′ CCTGATGGTGATGTCGGTCT 3′	3′ ACTAGCTCGGCTGCCTGAT 5′
mdtF	5′ CTGATGGTAGCGGCGTTTAT 3′	3′ GGCATACTCGGAACCAAAGA 5′
gapHD	5′ ACTTACGAGCAGATCAAAGC 3′	3′ AGTTTCACGAAGTTGTCGTT 5′
envR	5′ TAACTCCCGCAATGAAGGCT 3′	3′ CAGCATGGCGTAAGCAAGAC 5′

Table S5. Primers used for the determination of transcript levels
